# Broad-spectrum inflammasome inhibition by thiomuscimol

**DOI:** 10.1038/s41420-024-02238-2

**Published:** 2024-11-16

**Authors:** Marisa J. Anderson, Andreas B. den Hartigh, Wendy P. Loomis, Susan L. Fink

**Affiliations:** Department of Laboratory Medicine and Pathology, University of Washington, Seattle, Washington, USA

**Keywords:** Cell death, Immunology

## Abstract

Inflammasome formation, arising from pathogen or internal activating signals, is a key step in canonical pyroptosis, a gasdermin-mediated inflammatory cell death. Inhibition of pyroptosis has great clinical relevance due to its involvement in many different disease states. Current inhibitors of pyroptosis either only inhibit the final lytic step, which still allows inflammatory signal release, or only inhibit a single inflammasome, which does not account for inherent redundancy in activation of other inflammatory pathways. Here, we show that thiomuscimol, a structural analog of the lysis inhibitor muscimol, exhibits unique inhibitory activity upstream of plasma membrane rupture. We find that thiomuscimol inhibits inflammasome formation, as well as downstream caspase-1 activation, initiated by multiple pyroptotic signals, regardless of whether NLR recruitment of caspase-1 to the inflammasome relies on the ASC adapter protein. The ability of thiomuscimol to block multiple different inflammasomes opens the door for development of therapeutics with increased applications to broadly inhibit pyroptosis in multiple pathological settings.

## Introduction

Inflammasomes are innate immune signaling complexes that contribute to defense against infection [1], but can also initiate pathologic inflammation and cell death, contributing to disease [2]. Inflammasome activation is initiated by sensor proteins that detect molecular patterns and perturbations associated with pathogens and cellular damage [3]. Activated inflammasome proteins recruit pro-caspase-1, either directly via caspase recruitment domain (CARD) interactions, or through pyrin domain (PYD) interactions with the adapter protein ASC. The activated caspase-1 protease cleaves pro-interleukin-1β (IL-1β) and pro-IL-18 to produce active inflammatory cytokines. Caspase-1 also cleaves gasdermin D, allowing plasma membrane recruitment and oligomerization of the N-terminal pore-forming domain. The gasdermin pore facilitates secretion of proinflammatory IL-1β and IL-18 and initiates downstream events such as ninjurin-1 oligomerization and plasma membrane rupture leading to pyroptotic cell death [4].

Although inflammasomes are a crucial element of innate immune defense, the resulting inflammation and cellular loss due to pyroptosis have been implicated in initiation and progression of many disease states. One example is sepsis, where infection triggers exuberant inflammasome-mediated inflammation and cellular injury, which contribute to life-threatening organ dysfunction [5, 6]. Many pathogens activate multiple inflammasomes, creating overlapping layers of host defense that mitigate pathogen evasion mechanisms, but also creating significant challenges for therapeutic intervention [2]. Inflammasomes are also involved in multiple common diseases associated with sterile inflammation and cell death, including cardiovascular diseases, neurodegenerative conditions, and autoimmune diseases [7–10]. In addition, mutations in inflammasome components cause inherited autoinflammatory disorders with symptoms directly attributed to inflammasome activation [11].

Due to these associated pathogenic consequences, strategies to inhibit inflammasome activation and subsequent cytokine production and pyroptosis have been widely pursued. Much progress has been made in identifying inhibitors that target specific steps in the activation pathways of individual inflammasomes. For example, several new small molecule inhibitors, such as MCC950/CRID3 [12] or more recently, DVF890 [13, 14] have been found to specifically target and inhibit NLRP3 [12–14]. Both of these inhibitors specifically bind to the NLRP3 protein and are therefore not effective at inhibiting other inflammasomes [15]. Though specific inflammasome inhibition can certainly be beneficial, redundancy in inflammasome pathways may complicate therapeutic efficacy.

**Fig. 1:**
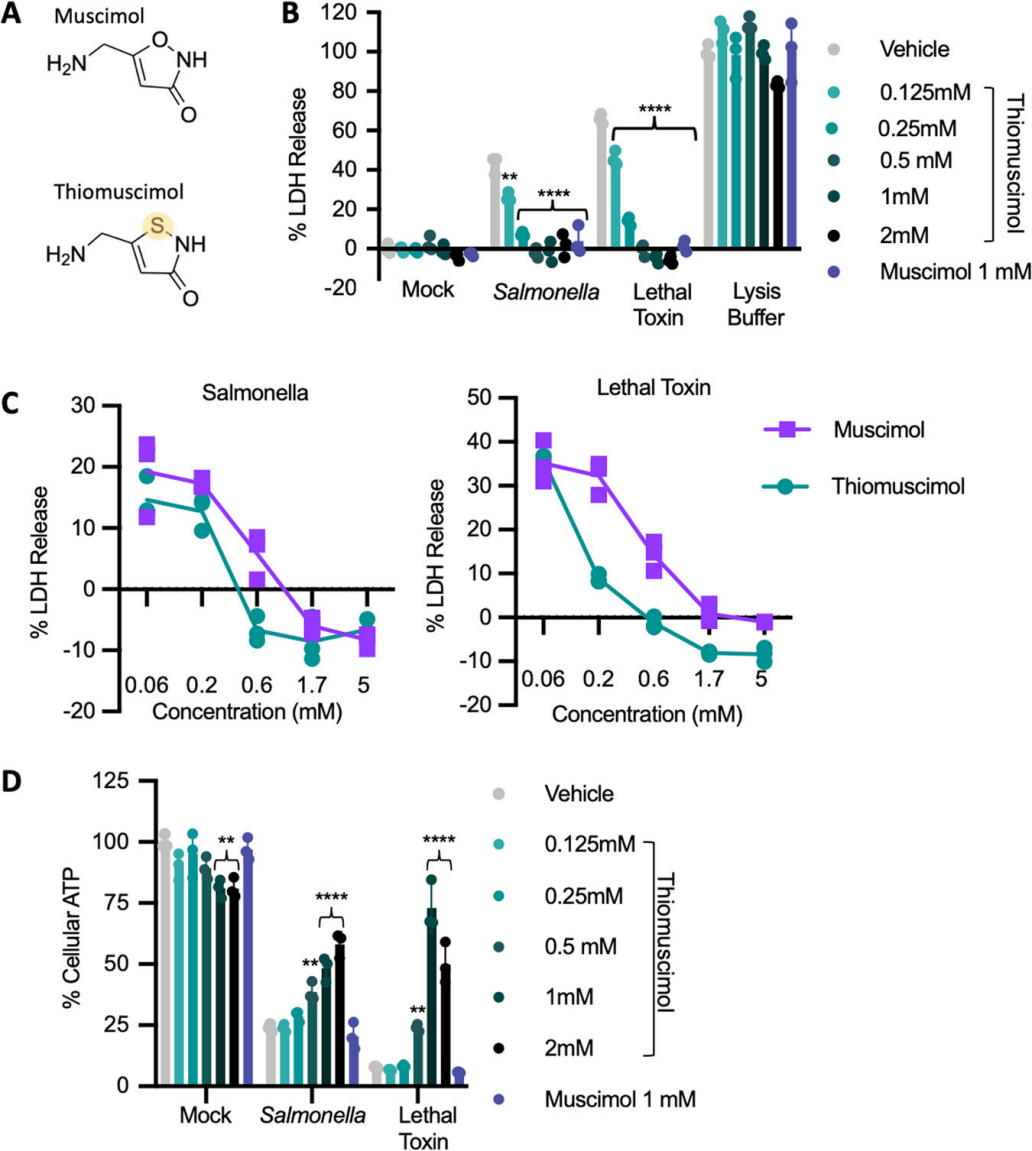
Both thiomuscimol and muscimol inhibit pyroptotic lysis, but thiomuscimol alone rescues cell viability. Structures of muscimol and thiomuscimol, with the single oxygen to sulfur replacement highlighted (**A**). Bone marrow-derived macrophages (BMDM) were treated with *Salmonella*, lethal toxin or lysis buffer in the presence of the indicated concentrations of thiomuscimol or muscimol (**B**–**D**). LDH released during pyroptotic lysis was measured (**B**, **C**). Viability was assessed by measuring cellular ATP content (**D**). Data are means±SD, *n* = 3 replicates, representative of two independent experiments. **P < 0.01, ***P < 0.001, ****P < 0.0001 (Ordinary one-way ANOVA with Dunnett’s multiple comparison test) indicates significance compared to vehicle control. Curly brackets indicate shared significance value.

Previously, we identified small molecule inhibitors which act downstream of inflammasome formation, and block plasma membrane rupture during pyroptosis [16]. One such molecule is muscimol, which we found blocks oligomerization of ninjurin-1, required for plasma membrane rupture downstream of gasdermin D pore formation [17]. To understand the molecular features enabling inhibition by muscimol, we examined a panel of muscimol analogs and found that most of the tested structural changes resulted in loss of protective activity [17]. Here, we characterize the activity of thiomuscimol, a structural analog of muscimol that differs only by a single oxygen to sulfur replacement within its carbon ring (Fig. 1A). Surprisingly, we found that thiomuscimol blocks pyroptotic events prior to plasma membrane rupture and acts via an entirely different mechanism compared to muscimol.

## Results

### Thiomuscimol, unlike muscimol, blocks pyroptotic events prior to plasma membrane rupture

The NLRC4 and NLRP1b inflammasomes are activated by *Salmonella* infection and anthrax lethal toxin treatment, respectively, resulting in pyroptosis with plasma membrane rupture. Consistent with our previous study [17], we observed that muscimol prevents plasma membrane rupture in *Salmonella*-infected or lethal toxin-treated pyroptotic macrophages, as assessed by measuring release of the large tetrameric cytoplasmic enzyme, lactate dehydrogenase (LDH) (Fig. 1B, C). To understand the structure-activity relationship for muscimol inhibition, we previously tested muscimol analogs and found that most molecular modifications eliminate protection against plasma membrane rupture. Thiomuscimol is molecularly very similar to muscimol, differing only by substitution of a single oxygen to sulfur within the carbon ring (Fig. 1A). Unlike the majority of muscimol analogs we tested previously, we found that thiomuscimol potently inhibited LDH release from both *Salmonella*-infected and lethal toxin-treated macrophages without affecting detection of LDH released from detergent-treated cells (Fig. 1B). Thiomuscimol matched muscimol in its inhibition of LDH release, with moderately increased potency in attaining inhibition at lower concentrations during lethal toxin treatment (Fig. 1C). We did not observe LDH release from cells treated with thiomuscimol alone, suggesting there was no toxicity at any of the tested concentrations (Fig. 1B).

Cytosolic ATP is thought to be lost through gasdermin D pores [18]. Thus, cell death, as defined by cessation of metabolic activity, occurs independently of plasma membrane rupture during pyroptosis. We confirmed our previous finding [17] that loss of cellular ATP in *Salmonella*-infected cells was not rescued by muscimol (Fig. 1D). We also observed the same result in response to lethal toxin treatment (Fig. 1D), consistent with the ability of muscimol to block plasma membrane lysis but not gasdermin D pore formation. In contrast to muscimol, thiomuscimol demonstrated a dose-dependent restoration of ATP content in pyroptotic cells, both in response to *Salmonella* infection and anthrax lethal toxin treatment (Fig. 1D), suggesting that thiomuscimol is also able to restore cell viability.

**Fig. 2:**
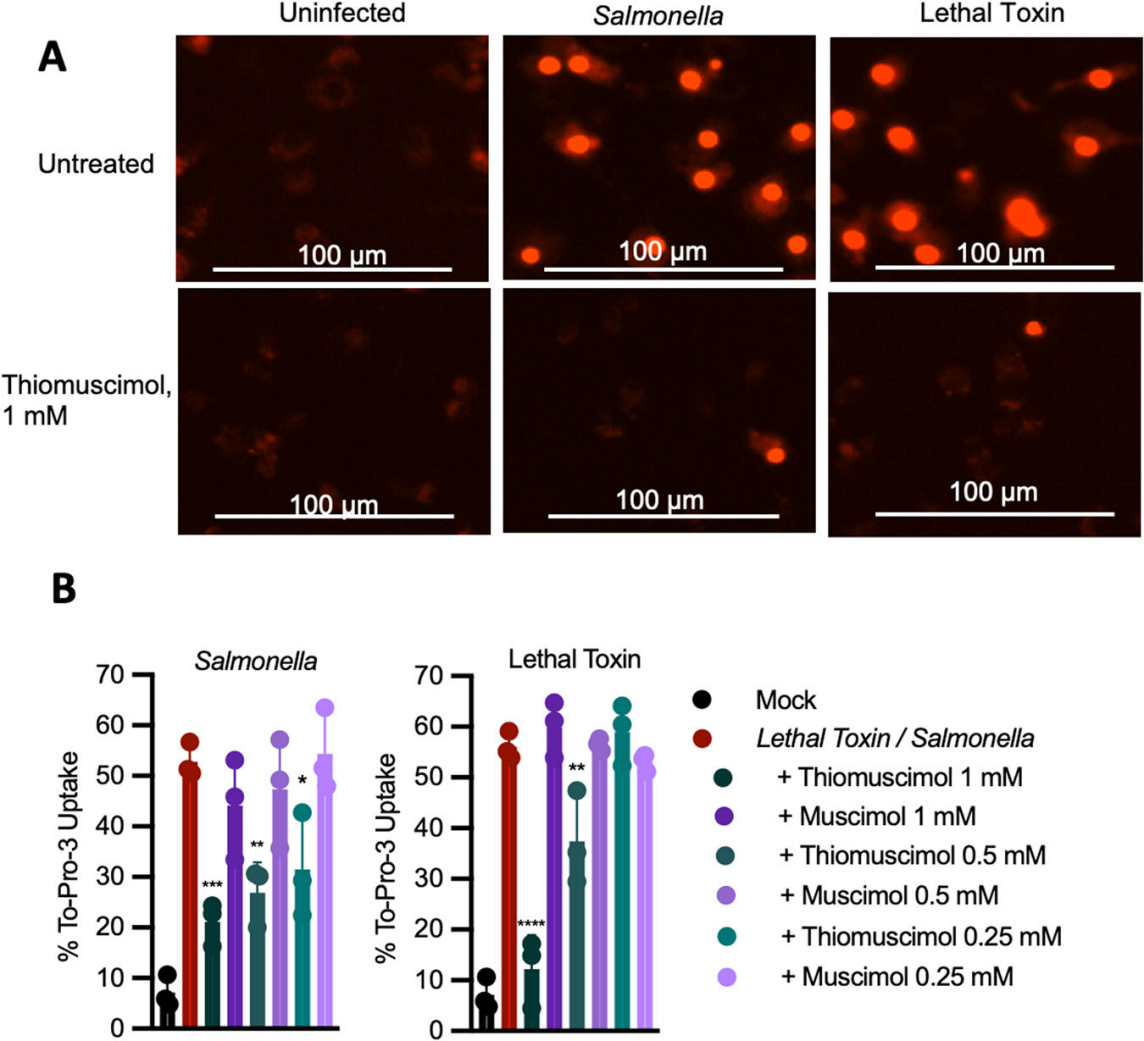
Thiomuscimol inhibits To-PRO-3 uptake in a dose-dependent manner. BMDM were treated with *Salmonella* or lethal toxin for a total of 2.5 h in the presence of the indicated concentrations of thiomuscimol or muscimol and uptake of the membrane-impermeant nuclear dye To-PRO-3 was assessed (**A**, **B**). Data are means ± SD, *n* = 3 replicates, representative of two independent experiments. *P < 0.05, **P < 0.01, ***P < 0.001, ****P < 0.0001 (Ordinary one-way ANOVA with Dunnett’s multiple comparison test) indicates significance compared to pyroptotic stimulus in the absence of thiomuscimol.

Upon inflammasome formation, active caspase-1 cleaves gasdermin D, releasing the N-terminal pore-forming domain which inserts into the plasma membrane. Gasdermin D pore formation allows influx of small molecules such as the membrane-impermeable fluorescent nucleic acid stain, To-PRO-3. Unlike muscimol, thiomuscimol inhibited To-PRO-3 uptake in both *Salmonella*-infected and lethal toxin-treated cells (Fig. 2A, B). Together, the preservation of cellular ATP content and inhibition of small dye uptake through the gasdermin D pore suggest that thiomuscimol acts earlier in the pyroptotic cascade than muscimol, which inhibits ninjurin-1-mediated membrane rupture [17].

### Thiomuscimol does not interfere with *Salmonella* effector translocation or lethal toxin entry

*Salmonella* utilizes type III secretion systems to deliver bacterial effector proteins into the cytoplasm of host cells [19]. Host cytosolic NAIP proteins recognize components of the type III secretion system, as well as inadvertently translocated bacterial flagellin, and activate caspase-1 via the NLRC4 inflammasome protein [20]. To determine whether thiomuscimol prevents NLRC4 inflammasome activation by interfering with bacterial effector translocation, we used *Salmonella* expressing the effector protein SspA fused to the catalytic domain of the CyaA adenylate cyclase toxin [21]. This fusion protein catalyzes the formation of cAMP in the presence of the cofactor calmodulin present in the host cytosol, and cAMP levels correlate with effector translocation. We found robustly increased cAMP levels in infected cells, that were unaffected by thiomuscimol (Sup Fig. 1a). This result indicates that thiomuscimol does not prevent translocation of *Salmonella* type III secretion system effector proteins into the host cell cytoplasm.

We next sought to determine whether thiomuscimol affects early events in the process of lethal toxin-induced pyroptosis. Anthrax lethal toxin is comprised of two proteins, protective antigen and lethal factor. Oligomerized protective antigen binds cellular receptors, initiating receptor-mediated endocytosis and translocation of lethal factor into the host cell cytoplasm [22]. Lethal factor is a protease that cleaves cellular targets including mitogen-activated protein kinase kinase 3 (MKK3) [23]. To determine whether proteolysis of cellular targets by lethal factor is affected by thiomuscimol, we assessed MKK3 cleavage via Western blot (Sup Fig. 1b). We found loss of full length MKK3 in lethal toxin-treated cells and the appearance of cleaved MKK3. Cleavage of MKK3 was not prevented by thiomuscimol (Sup Fig. 1b). Together, these findings indicate that thiomuscimol does not interfere with *Salmonella* effector translocation or lethal toxin entry, but rather blocks gasdermin D pore formation and pyroptotic plasma membrane rupture in response to these two stimuli.

In addition to caspase-1, ASC-containing inflammasomes can recruit and activate caspase-8, the canonical initiator of the extrinsic pathway of apoptosis [24, 25]. In wildtype cells, caspase-1 and gasdermin D-dependent pyroptosis limits caspase-8 activation by ASC-containing inflammasomes. However, when caspase-1 is absent or enzymatically inactivated, inflammasome activation leads to caspase-8 and caspase-3-mediated apoptosis [24, 25]. To determine whether thiomuscimol redirects *Salmonella*-infected macrophages toward caspase-3-mediated apoptosis, we examined caspase-3 activity using a fluorescent substrate cleavage assay. Although we detected caspase-3 activity in response to the apoptosis-inducer staurosporine, thiomuscimol did not activate caspase-3 in *Salmonella*-infected macrophages (Sup Fig. 2). This result indicates that thiomuscimol inhibits pyroptosis, without redirecting cells towards apoptosis, suggesting that multiple cell death pathways triggered by ASC-containing inflammasomes are blocked.

### Thiomuscimol inhibits activation of multiple inflammasomes

**Fig. 3:**
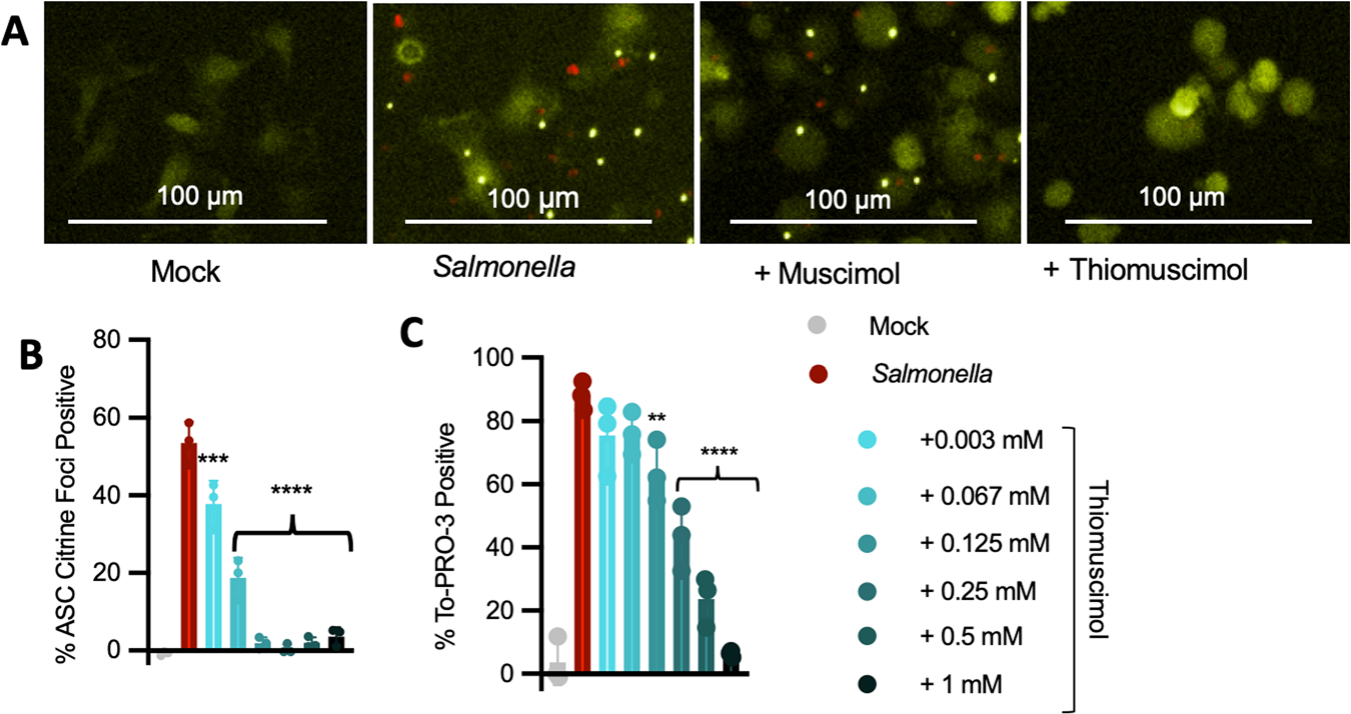
Thiomuscimol inhibits ASC speck formation and To-PRO-3 uptake in a dose-dependent manner. ASC-citrine expressing BMDM were infected with *Salmonella* for 90 min in the presence of muscimol or thiomuscimol as indicated. Localization of the inflammasome adapter ASC (yellow) and uptake of the small membrane-impermeant nuclear dye To-PRO-3 (red) were assessed (**A**–**C**). Data are means ± SD, *n* = 3 replicates, representative of two independent experiments. *P < 0.05, **P < 0.01, ***P < 0.001, ****P < 0.0001 (Ordinary one-way ANOVA with Dunnett’s multiple comparison test) indicates significance compared to infection in the absence of thiomuscimol. Curly brackets indicate shared significance value.

ASC oligomerization can be detected using live cell microscopy in primary macrophages expressing ASC fused with the fluorescent protein citrine [26]. The ASC-citrine protein is diffusely distributed throughout the cytoplasm of uninfected cells but redistributes during *Salmonella* infection into a single large focus or “speck” per cell (Fig. 3A). Consistent with our previous results [17], we found that muscimol had no effect on ASC speck formation in *Salmonella*-infected macrophages (Fig. 3A). In contrast, thiomuscimol prevented ASC speck formation (Fig. 3A) in a dose-dependent manner (Fig. 3B), along with To-PRO-3 uptake (Fig. 3C). To determine whether the protective effects of thiomuscimol could be further optimized, we treated macrophages with thiomuscimol at multiple concentrations one hour prior to *Salmonella* infection. We found that thiomuscimol pretreatment did not significantly enhance inhibition of ASC-citrine specks or To-PRO-3 uptake (Sup Fig. 3a, b), and was thus not included in further experiments.

**Fig. 4:**
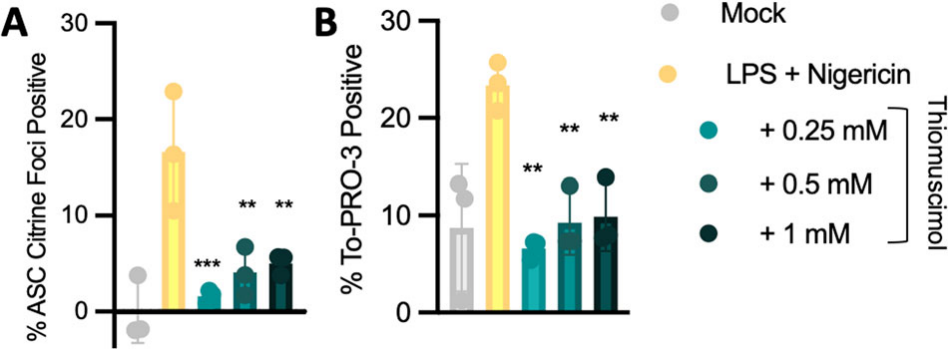
Thiomuscimol inhibits NLRP3 induced ASC speck formation and To-PRO-3 uptake. ASC-citrine expressing BMDM were primed with LPS and treated with nigericin to activate NLPR3 in the presence of thiomuscimol as indicated. ASC speck formation and uptake of the small membrane-impermeant nuclear dye To-PRO-3 were assessed (**A**, **B**). Data are means ± SD, *n* = 3 replicates, representative of two independent experiments. *P < 0.05, **P < 0.01, ***P < 0.001 (Ordinary one-way ANOVA with Dunnett’s multiple comparison test) indicates significance compared to NLRP3 activation in the absence of thiomuscimol.

Because different inflammasomes have differing methods of activation and assembly, we sought to understand if thiomuscimol also inhibits the widely-studied NLRP3 inflammasome. NLRP3 is activated via a two-step process with a priming stimulus needed prior to NLR activation [27]. We primed ASC-citrine expressing macrophages with LPS and then activated NLRP3 with nigericin. ASC-citrine distribution and To-PRO-3 uptake were monitored using live cell microscopy. We found that thiomuscimol prevented both ASC speck formation and To-PRO-3 uptake in response to nigericin (Fig. 4A, B), indicating inhibition of the NLRP3 inflammasome.

**Fig. 5:**
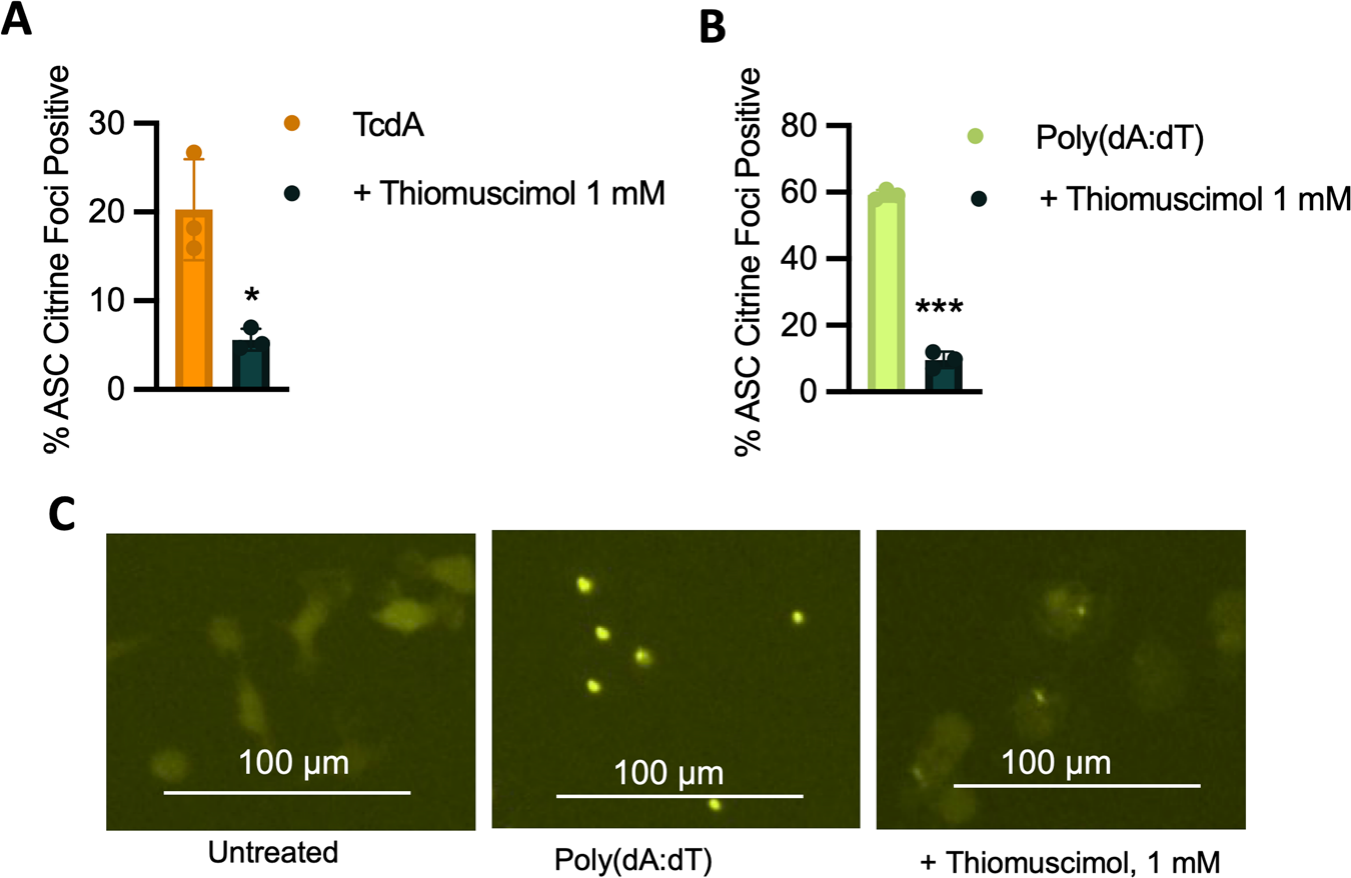
Thiomuscimol inhibits Pyrin and AIM2 induced ASC speck formation. ASC-citrine expressing BMDM were primed with LPS (500 ng/mL, 3 h) and treated with TcdA or poly(dA:dT) for 3 h to activate pyrin or AIM2 respectively in the presence of thiomuscimol as indicated. ASC speck formation was kinetically measured over time to monitor inflammasome formation (**A**–**C**). Data are means ± SD, *n* = 3 replicates, representative of two independent experiments. *P < 0.05, **P < 0.01, ***P < 0.001 (Unpaired T-test) indicates significance compared to pyrin activation in the absence of thiomuscimol.

Thus far, our results suggest that thiomuscimol inhibits activation of the NLRC4, NLRP1b and NLRP3 inflammasomes. These three inflammasome sensors belong to the NLR protein family and share similar domain structures, including the presence of a leucine-rich repeat (LRR) domain. To further examine the effects of thiomuscimol on differing inflammasomes, we sought to understand if thiomuscimol inhibits inflammasomes that are outside the NLR family. Pyrin is an inflammasome sensor protein that detects pathogen infection via toxin-mediated inactivation of RhoA GTPases [28]. We activated the pyrin inflammasome by treating ASC-citrine expressing macrophages with the *Clostridium difficile* toxin, TcdA. We observed that ASC speck formation in response to pyrin activation with TcdA was inhibited by thiomuscimol (Fig. 5A). We additionally tested AIM2, which recognizes cytoplasmic double-stranded DNA [29]. We transfected ASC-citrine expressing macrophages with poly(dA:dT) to activate AIM2 and observed ASC speck formation that was inhibited by thiomuscimol (Fig. 5B). However, upon closer examination, we observed small, dim ASC spots present in poly(dA:dT) transfected macrophages in the presence of thiomuscimol (Fig. 5C), which did not meet the size and intensity thresholds of standard ASC specks for quantitative image analysis. This morphology was distinctly different from the uniform condensation of all cytoplasmic ASC into a large, bright speck observed in the absence of thiomuscimol (Fig. 5C). This finding suggested that some aspects of AIM2 inflammasome formation may be unaffected by thiomuscimol, although the ability of the AIM2-induced ASC speck to form in its entirety is blocked. Together, these findings suggest that thiomuscimol inhibits aspects of activation of multiple inflammasomes.

### Thiomuscimol activity is reversible and not recapitulated by other PDI inhibitors

**Fig. 6:**
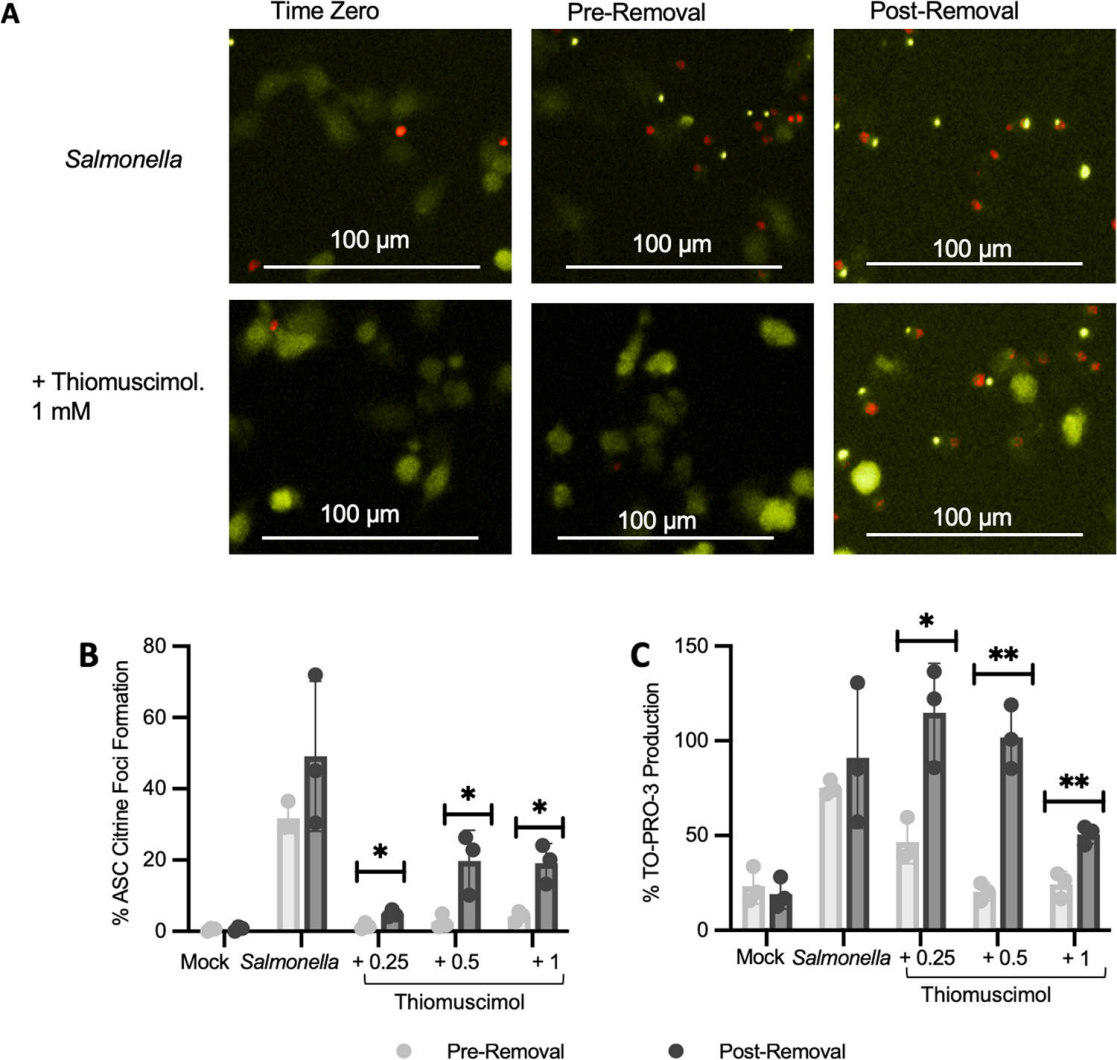
Inhibition of inflammasome activation by thiomuscimol is reversible. ASC-citrine expressing BMDM were infected with *Salmonella* for one hour in the presence of thiomuscimol as indicated. The cell culture medium was removed and replaced with fresh medium containing To-PRO-3 but no thiomuscimol and cells were imaged for an additional hour. Localization of the inflammasome adapter ASC (yellow) and uptake of the small membrane-impermeant nuclear dye To-PRO-3 (red) were assessed before and after removal of the cell culture medium (**A**–**C**). Data are means ± SD, *n* = 3 replicates, representative of two independent experiments. *P < 0.05, **P < 0.01, ***P < 0.001 (unpaired t-test).

To further understand the mechanism of thiomuscimol, we tested whether thiomuscimol-mediated inhibition is reversible. We infected ASC-citrine expressing macrophages with *Salmonella* in the presence or absence of thiomuscimol and monitored ASC-citrine speck formation and To-PRO-3 uptake via fluorescence microscopy (Fig. 6A–C). After one hour of infection, we removed the cell culture medium and incubated for an additional hour in fresh medium containing To-PRO-3 without thiomuscimol to determine if the inhibitory effects of thiomuscimol would dissipate. Post-removal, there was a significant increase in both ASC-citrine speck formation (Fig. 6A, B) and To-PRO-3 uptake (Fig. 6A, C) at all tested concentrations. The rebound in ASC-citrine speck formation and To-PRO-3 uptake after thiomuscimol removal suggests that thiomuscimol inhibition of inflammasome formation and pyroptosis is a reversible phenomenon. These findings further confirm that thiomuscimol does not damage cells or permanently alter processes essential to inflammasome activation and pyroptosis.

**Fig. 7:**
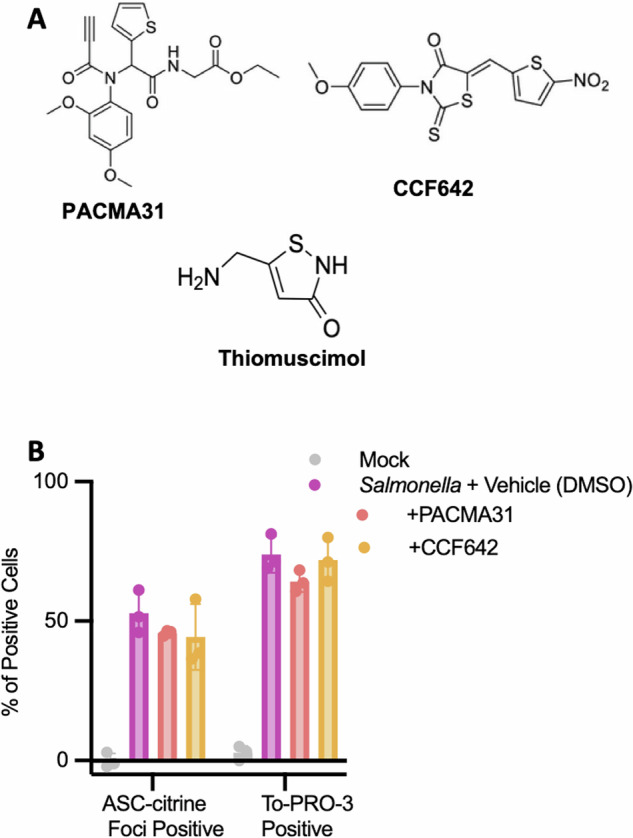
PDI inhibition is not sufficient to inhibit inflammasome activation. Structures of PDI inhibitors PACMA31 and CCF642 (**A**). ASC-citrine expressing BMDM were infected with *Salmonella* in the presence of the indicated PDI inhibitors or 1% DMSO vehicle control. ASC speck formation and uptake of the small membrane-impermeant nuclear dye To-PRO-3 were assessed (**B**). Data are means ± SD, *n* = 3 replicates, representative of two independent experiments. No significant difference was found between either drug condition and the infected vehicle control (One-way ANOVA, Tukey’s post-hoc analysis).

We next researched previously identified targets of thiomuscimol that could contribute to its activity. Thiomuscimol, but not muscimol, has been described to inhibit the enzymatic activity of protein disulfide isomerase (PDI) [30]. This observation suggested the hypothesis that thiomuscimol may block inflammasome activation and pyroptosis via inhibiting PDI. To test this hypothesis, we identified two other small molecule inhibitors of PDI, PACMA 31 [31, 32] and CCF642 [33, 34] (Fig. 7A). We treated *Salmonella-*infected macrophages with these inhibitors at concentrations described to inhibit PDI and assessed ASC-citrine speck formation and To-PRO-3 uptake using live cell microscopy (Fig. 7B). We found that neither PDI inhibitor significantly inhibited ASC-citrine speck formation or To-PRO-3 uptake (Fig. 7B). This finding indicates that inhibition of inflammasome activation and pyroptosis is not recapitulated by other PDI inhibitors.

### ASC-independent inflammasome activation is blocked by thiomuscimol

Inflammasomes recruit and activate caspase-1 via the speck-forming protein ASC, but both NLRC4 and NLRP1b contain CARD domains that can directly recruit and activate caspase-1, independently of ASC specks [35, 36]. In this setting, caspase-1-mediated gasdermin D processing and pore formation can occur independently of ASC [37]. We found that thiomuscimol inhibits ASC speck formation (Figs. 3 and 4), but also pore-mediated To-PRO-3 uptake (Figs. 2 and 4), suggesting that ASC-independent caspase-1 activation may also be blocked.

**Fig. 8:**
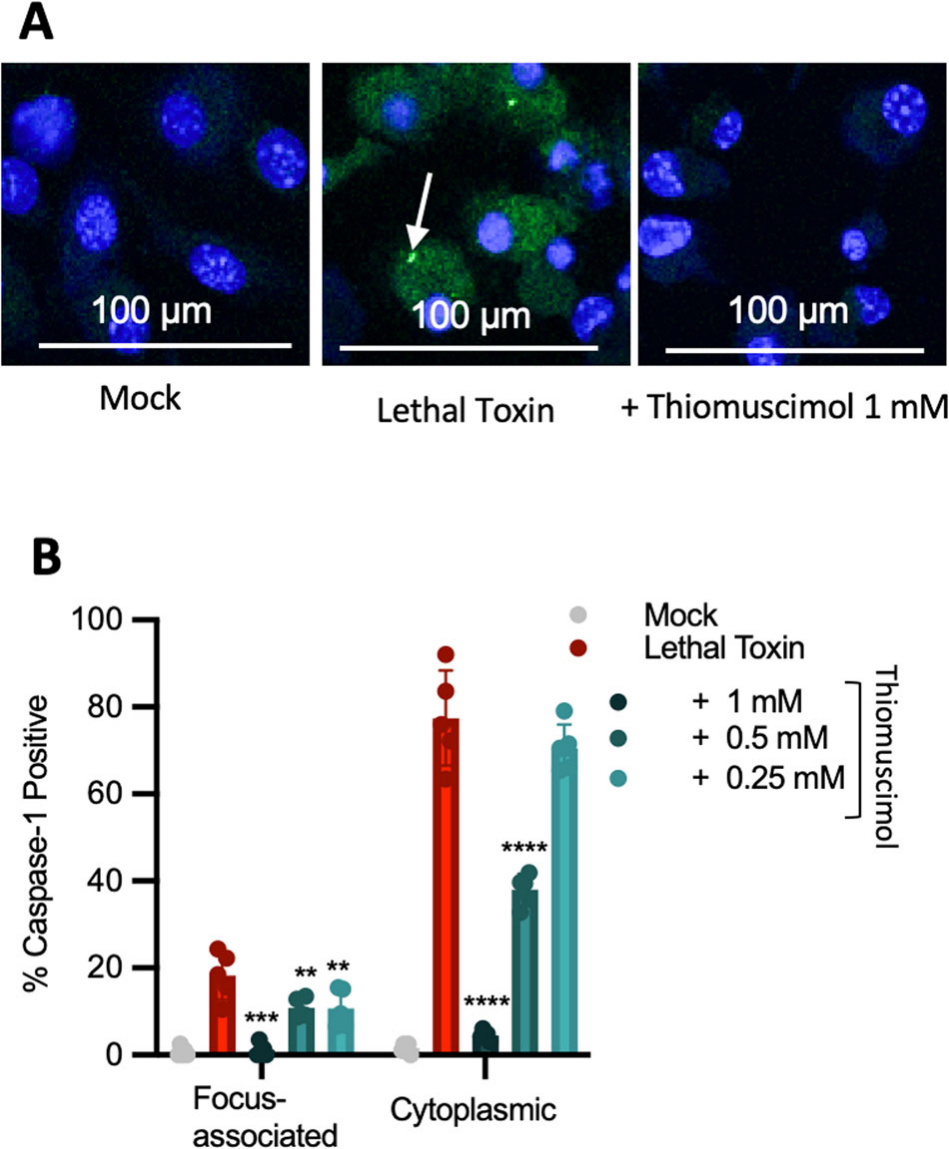
Thiomuscimol inhibits both speck-localized and diffuse cytoplasmic caspase-1 activation. BMDM were treated with lethal toxin for three hours in the presence of thiomuscimol as indicated. Caspase-1 activation was detected using FAM-YVAD-FMK (green) and nuclei were counterstained (blue) (**A**). The arrow points to an example of speck-localized active caspase-1. The percentage of cells containing speck-localized and diffuse cytoplasmic active caspase-1 were quantified individually (**B**). Data are means ± SD, *n* = 5 visual fields, representative of two independent experiments. *P < 0.05, **P < 0.01, ***P < 0.001, ****P < 0.0001 (Ordinary one-way ANOVA with Dunnett’s multiple comparison test) indicates significance compared to lethal toxin treatment in the absence of thiomuscimol.

**Fig. 9:**
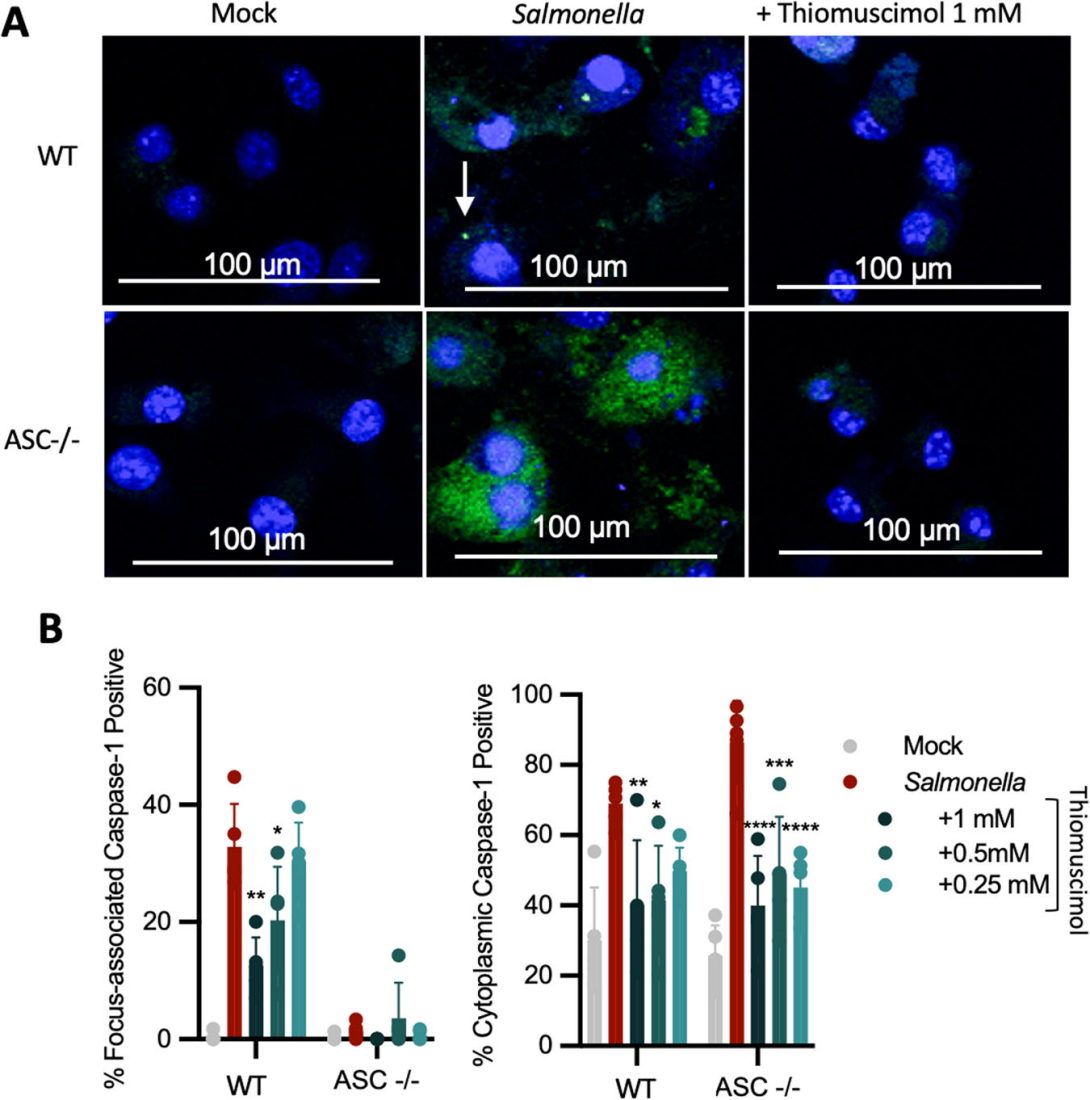
Thiomuscimol inhibits ASC-dependent and ASC-independent caspase-1 activation. Wild type C57BL/6 J (WT) and ASC −/− BMDM were infected with *Salmonella* for two hours in the presence of thiomuscimol as indicated. Caspase-1 activation was detected using FAM-YVAD-FMK (green) and nuclei were counterstained (blue) (**A**). The arrow points to an example of speck-localized active caspase-1. The percentage of cells containing speck-localized and diffuse cytoplasmic active caspase-1 were quantified individually (**B**). Data are means ± SD, *n* = 3-5 visual fields, representative of two independent experiments. *P < 0.05, **P < 0.01, ***P < 0.001, ****P < 0.0001 (Ordinary one-way ANOVA with Dunnett’s multiple comparison test) indicates significance compared to infection in the absence of thiomuscimol.

To directly assess caspase-1 activation, we used the FAM-YVAD-FMK caspase-1 activity probe, which fluorescently labels active caspase-1 [38]. We found active caspase-1 localized to specks and diffusely throughout the cytoplasm in lethal toxin-treated cells (Fig. 8A). Thiomuscimol inhibited both speck-localized and diffuse cytoplasmic caspase-1 activation in a dose-dependent manner (Fig. 8B). To further examine the ability of thiomuscimol to inhibit cytoplasmic caspase-1 activation, we utilized ASC-deficient macrophages. We infected ASC-deficient and wildtype macrophages with *Salmonella* in the presence or absence of thiomuscimol at multiple concentrations and stained for active caspase-1 using the FAM-YVAD-FMK probe. As expected, we found both speck-localized and diffuse cytoplasmic active caspase-1 in infected wildtype cells (Fig. 9A- top row). While staining of diffuse active caspase-1 was observed in the absence of ASC, speck-localized caspase-1 activation was not detected in *Salmonella*-infected, ASC-deficient macrophages (Fig. 9A- bottom row). We observed significant inhibition of both speck-localized and diffuse cytoplasmic active caspase-1 by thiomuscimol (Fig. 9B). Taken together, these results indicate that thiomuscimol acts as a broad-spectrum inflammasome inhibitor, with inhibitory effects that are not reliant on an inflammasome-specific context.

## Discussion

Although inflammasomes are essential for innate host defense, pyroptotic cell death and its inflammatory consequences are linked to a growing number of prevalent diseases, making inflammasome inhibition of great clinical relevance [14]. In this study, we identified thiomuscimol as a unique inhibitor of the NLRP1b, NLRP3 NLRC4, pyrin, and AIM2 inflammasomes, with a mechanism of action independent of the inflammasome adapter, ASC. This property is distinct from previously identified small molecules that block specific inflammasomes, such as MCC950/CRID3 which selectively targets NLRP3 and has no effect on NLRC4 or NLRP1b [39]. Although the ASC oligomerization inhibitor MM01 blocks multiple ASC-dependent inflammasomes [40], we found that thiomuscimol additionally prevents ASC-independent inflammasome-mediated caspase-1 activation and pyroptotic death. Redundancy within cell death pathways means that therapeutic efforts to block downstream caspase-1 or gasdermin D activation may only divert cells to other pathways of death [24, 41] and not ultimately prevent pathologic sequelae. We found that thiomuscimol prevents both inflammasome-induced pyroptosis and apoptosis, and at least partially restores ATP content, suggesting a unique and potentially beneficial effect to preserve cell viability.

Direct inhibition of caspase-1 has been of recent clinical interest, though many of the discovered inhibitors are peptide based or peptidomimetic and have presented challenges such as poor ability to cross membranes, or high levels of adverse effects [42]. Prior work has shown that enzymatically inactive caspase-1 (similar to caspase-1−/−) results in an alternate cell death pathway, caspase-8 mediated apoptosis [43, 44], and in fact may still activate inflammation through the RIP-2 TNF-α pathway [45]. We found that thiomuscimol does not redirect to apoptosis, and that thiomuscimol inhibits caspase-1 whether or not the inflammasome interaction was ASC-reliant. Taken together, our results suggest that thiomuscimol inhibits a common aspect of inflammasome activation, rather than direct inhibition of caspase-1 enzymatic activity.

Muscimol is almost identical in structure to thiomuscimol, but the findings presented here and in our previous study [17] demonstrate that muscimol has no effect on inflammasome activation. Similarly, thiomuscimol, but not muscimol, inhibits PDI enzymatic activity [30]. However, our finding that other PDI inhibitors did not block inflammasome activation suggests that PDI inhibition alone may not be the mechanism by which thiomuscimol blocks inflammasomes. Thiomuscimol and muscimol have similar activities as neuronal GABA_A_ receptor agonists [46], but neither muscimol [17] nor GABA itself [16] inhibit inflammasome activation and pyroptosis, demonstrating that GABA_A_ receptor activity is not sufficient to inhibit inflammasome activation. Identifying the direct target(s) of thiomuscimol that mediate inflammasome inhibition would be an important avenue for future study. We found that thiomuscimol is more potent than muscimol at preventing plasma membrane rupture during pyroptosis, but the millimolar concentrations required for thiomuscimol are still quite high from a pharmacologic perspective. This, together with the known activities on PDI and GABA_A_ receptors indicates that developing inhibitors specific for the relevant target(s) will be an important future direction.

Muscimol differs from thiomuscimol only by substitution of a single oxygen to sulfur within the carbon ring, suggesting that the presence of sulfur in place of oxygen is a critical molecular determinant for the ability to inhibit inflammasome activation. In our previous examination of muscimol and structurally related compounds, we tested non-sulfated muscimol analogs and did not find any that act like thiomuscimol to inhibit inflammasome activation [17], indicating that inflammasome inhibition is not recapitulated by these other molecular substitutions. Sulfur has a larger atomic radius and lower electronegativity than oxygen, imparting distinct chemical properties. Sulfur-containing compounds exhibit diverse biological activities, including some that act as covalent inhibitors [47]. Further study is necessary to better define the biochemical basis for inflammasome inhibition, although we hypothesize that thiomuscimol may not act via irreversible or covalent target binding because we observed a rebound in ASC speck formation and To-PRO-3 uptake after thiomuscimol removal.

The NLRC4, NLRP1b, NLRP3, pyrin and AIM2 and inflammasomes are each activated via unique signals, yet remarkably these inflammasomes are all affected by thiomuscimol. We found that thiomuscimol does not interfere with *Salmonella* effector translocation or lethal toxin target proteolysis, indicating that initiation of these inflammasome activating signals is unaffected. Our finding of small, dim ASC spots in poly(dA:dT) transfected macrophages in the presence of thiomuscimol also suggests that initial AIM2 and ASC recruitment to cytoplasmic DNA polymers may be unaffected. Emerging data have revealed that inflammasome activation pathways involve multiple layers of regulation that could be targeted by small molecule inhibitors. For example, NLRP3 activation involves several post-translational modifications, including phosphorylation, acetylation and deubiquitination [39]. Whether there are common post-translational modifications or other regulatory mechanisms that are shared by multiple inflammasomes and could be targeted by thiomuscimol remains unclear and warrants further study. In addition, the specific biochemical processes mediating inflammasome protein oligomerization are not clear and there may be shared aspects between inflammasomes. Overall, further study will be needed to understand the detailed molecular mechanism underlying thiomuscimol’s activity.

Inflammasomes and pyroptosis are involved in the pathogenesis of leading global causes of mortality, including cardiovascular disease [48], infections [49, 50], and neurodegenerative diseases [51, 52]. The pathophysiology of these diseases is complex and multiple inflammasomes have been implicated. Thus, a broad-spectrum inflammasome inhibitor with activity like thiomuscimol could have many therapeutic applications. Thiomuscimol itself may not be an ideal therapeutic candidate due to the relatively high concentrations required and its other biologic activities. However, future characterization of thiomuscimol analogs could provide insights into the structure-activity relationship and reveal molecules with improved potency and specificity. Together, our findings demonstrate that broad-spectrum inflammasome inhibition is feasible with a single molecule and understanding the mechanism of action may reveal unexpected insights into shared molecular features underlying activation of multiple inflammasomes.

## Materials and methods

### Cell culture

Bone marrow-derived macrophages (BMDM) were cultured from the following strains of mice: BALB/cJ (Strain #:000651), C57BL/6 J (Strain #:000664), and C57BL/6 J ASC-citrine (Strain #:030744), all purchased from Jackson Laboratories. ASC −/− mice were a kind gift from Genentech. Bone marrow cells were isolated and pooled from male and female mice. Knockout mouse genotype was confirmed by PCR analysis. BMDM were grown for 7 days at 37 °C with 5% CO_2_ in Dulbecco’s Minimal Essential Medium (DMEM) supplemented with 10% Serum Plus II (Sigma), 5 mM Hepes, 0.05 mM β-mercaptoethanol, 100 U/mL penicillin and streptomycin, and 30% conditioned medium from L929 cells (ATCC Cat. No CCL-1, RRID:CVCL_0462). Additional medium was provided on day 3 or 4. Cells were collected with PBS containing 1 mM EDTA, resuspended and plated in supplemented medium without phenol red containing 5% Serum Plus II. As primary cells were used immediately after differentiation and not maintained in sub-culture, cell line validation and mycoplasma testing were not performed.

### Pyroptosis inducers and reagents

For activation of the NLRC4 inflammasome, late-log cultures of *Salmonella enterica* serovar Typhimurium strain SL1344 (a kind gift from Dr. Brad T. Cookson, University of Washington) were grown in Luria-Bertani broth containing 0.3 M NaCl with shaking at 37 °C. Bacteria were washed, resuspended in PBS, and added at a multiplicity of infection of 10 bacteria per macrophage for 90 minutes (unless otherwise indicated). For NLRP1b activation, BMDM from BALB/cJ mice were treated for two hours (unless otherwise indicated) with 1 µg/mL anthrax lethal toxin, prepared as a 1:1 mix of protective antigen and lethal factor (List Biological Labs). For NLRP3 activation, BMDM from C57BL/6 J mice were primed with 100 ng/mL of LPS (List Biological Labs) for three hours, followed by addition of 15 µM nigericin (Cayman Chemicals) for an additional three hours. For pyrin activation, BMDM from C57BL/6 J mice were primed with 500 ng/mL of LPS (List Biological Labs) for three hours, followed by addition of Toxin A (TcdA) from *Clostridium difficile* (2 µg/mL) (List Biological Labs). For AIM2 activation, BMDM from C57BL/6 J mice were primed with 500 ng/mL of LPS (List Biological Labs), before transfection of 2 µg/mL poly (dA:dT) (Invivogen) at a 1:25 ratio of DNA to lipofectamine (µg/µL) utilizing Lipofectamine 2000 + OptiMEM, as described [53]. Thiomuscimol (Santa Cruz Biotechnology and Cayman Chemicals) and muscimol hydrobromide (Sigma Aldrich) were added at specified concentrations, at the same time as the pyroptosis inducer, unless otherwise specified. The PDI inhibitors PACMA31 and CCF642 (Cayman Chemicals) were reconstituted in 100% DMSO and stored as frozen aliquots at −20°C before use. Each were diluted to target concentrations of 5 µM for CCF642 and 2.25 µM for PACMA31 in cell culture medium, resulting in a final DMSO concentration of 1%.

### Lactate dehydrogenase (LDH) release assay

Plasma membrane rupture was measured using the CytoTox 96 Non-Radioactive Cytotoxicity Assay kit (Promega) according to the manufacturer’s protocol. Maximum LDH release was obtained by using the provided detergent-based lysis buffer. OD490 was measured using a Spectramax M3 plate reader. LDH release was calculated as: 100 x (experimental LDH – spontaneous LDH)/(maximum LDH-spontaneous LDH).

### Cellular ATP measurement

Cellular ATP content was assessed using the luminescent CellTiter-Glo 2.0 Cell Viability Assay kit (Promega) according to the manufacturer’s protocol.

### Live cell fluorescence microscopy

BMDM were seeded in optical 96 well plates and treated with pyroptosis inducers. To assess ASC speck formation, we used cells from ASC-citrine fusion protein expressing mice. Membrane impermeable nuclear dye To-PRO-3 (1 µM) was added to assess pyroptotic pore formation. ASC speck formation and To-PRO-3 uptake were observed and quantified using a Cytation1 imaging system running Gen5 version 3.11 (Biotek).

### Bacterial type III secretion system effector translocation assay

Translocation of the SspA-Cya fusion was assessed, as previously described [21]. BMDM from C57BL/6 J mice were infected with *Salmonella* expressing the *Salmonella* pathogenicity island 1 (SPI1)-encoded type III secretion system effector protein SspA fused to Cya for 90 minutes using bacteria grown under SPI-1 inducing conditions. cAMP levels were determined using the Direct cAMP ELISA kit (Enzo Life Sciences).

### Western blot

BMDM from BALB/cJ mice were treated for 2 h with 1 µg/mL anthrax lethal toxin in medium containing 20 mM glycine (VWR), in the presence or absence of thiomuscimol. In-well lysis and protein extraction was performed with Protein Extraction Reagent Type 4 (MilliporeSigma) with added HALT protease Inhibitor Cocktail (ThermoFisher), and phenylmethylsulfonyl fluoride (PMSF) protease inhibitor (ThermoFisher). Cell lysates were frozen at −80 °C before testing. Thawed lysates were mixed with loading buffer (Laemmli buffer + 10% β-mercaptoethanol) and heated at 95 °C for 10 min under reducing conditions. Proteins were separated by SDS-PAGE using any kD TGX stain-free gels (Bio-Rad) and transferred to PVDF membranes. Vinculin was detected with a mouse monoclonal antibody (sc-73614, Santa Cruz Biotechnology) and goat anti-mouse RD680 secondary antibody (LiCor). MEK3 was detected with rabbit monoclonal MEK3 antibody (#8535 s, Cell signaling technologies) and donkey anti-rabbit secondary antibody IR Dye 680 RD (LiCor). Blots were scanned on a Licor Odyssey.

### Caspase-3 activity assay

After pyroptosis induction, caspase-3 activity was measured using the SensoLyte Homogenous AMC Caspase-3/7 Fluorometric Assay kit (Anaspec) according to the manufacturer’s protocol. Cells were treated with staurosporine (Sigma Aldrich) at 1 µM for 90 min to induce apoptosis.

### Fluorescence microscopy

BMDM were seeded on coverslips coated in poly-d-lysine in 24 well plates and treated with pyroptosis inducers in medium containing 5 mM glycine. Active caspase-1 was detected with the FAM-YVAD-FMK activity probe (ImmunoChemistry Technologies) according to the manufacturer’s protocol. Cells were fixed and permeabilized with BD CytoFix/CytoPerm and counterstained with To-PRO-3. Images were obtained using a Leica SP8X confocal system running the LasX imaging software using a ×63 NA 1.4 oil immersion objective, and the percent of positive cells was determined using 3–5 high powered fields for each condition per experiment.

### Statistics and reproducibility

Sample size was based on experience performing similar experiments, and no sample size calculations were performed. Appropriate statistical analysis was performed in Prism (GraphPad software, v.10). The statistical tests used and the number of replicates are indicated in each figure legend, and all data are presented as mean ± SD unless otherwise specified. All figures were prepared in Prism (GraphPad software, v.10).

## Supplementary information

Original Data

Supplemental Figure

## Data Availability

The uncropped Western blot for Fig. S1b is provided in Fig S4. All the data can be found either in the main text or the supplementary materials.
