# Supplementary figures and images for: Broad-spectrum inflammasome inhibition by thiomuscimol
Source: Cell Death Discov. 2024 Nov 16;10:470. doi: 10.1038/s41420-024-02238-2 (PMC11569204; doi:10.1038/s41420-024-02238-2)

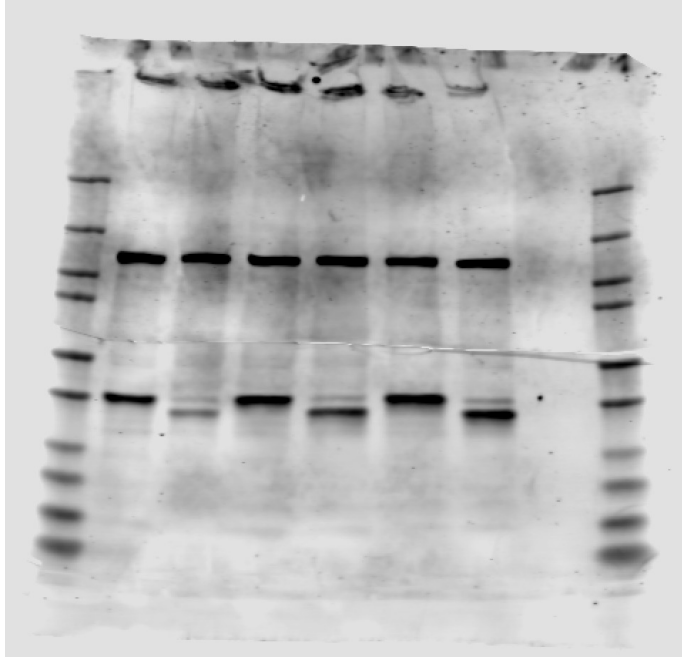

**Supplemental Figure 4. Complete western blot**  
Uncropped western blot for supplemental figure 1.

Supplement: Supplementary file 1 — Original Data [file 41420_2024_2238_MOESM1_ESM.pdf]
