# Supplementary material for: Broad-spectrum inflammasome inhibition by thiomuscimol
Source: Cell Death Discov. 2024 Nov 16;10:470. doi: 10.1038/s41420-024-02238-2 (PMC11569204; doi:10.1038/s41420-024-02238-2)
Supplement: Supplementary file 2 — Supplemental Figure [file 41420_2024_2238_MOESM2_ESM.pdf]

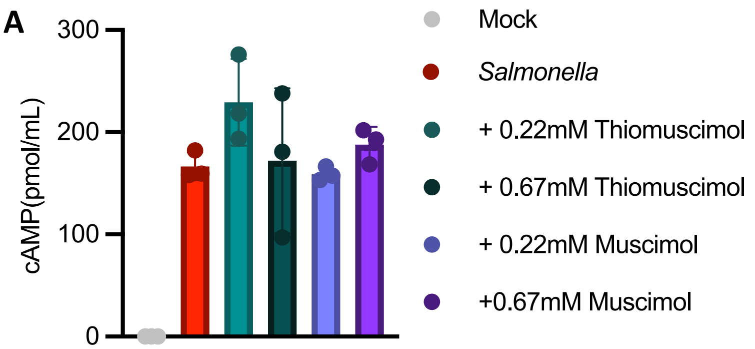

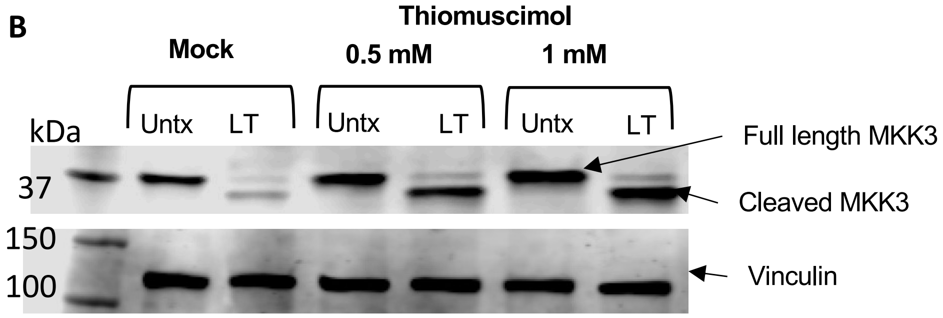

Supplemental Figure 1. Thiomuscimol does not inhibit *Salmonella* effector translocation or lethal toxin entry
BMDM were infected with *Salmonella* expressing a type III secretion effector protein fused to adenylate cyclase in the presence of the indicated concentrations of thiomuscimol or muscimol. Effector translocation was assessed by measuring cellular cAMP levels (A). BMDM were treated with lethal toxin in the presence of thiomuscimol as indicated and cleavage of MKK3 was determined by Western blot (B). Data are means +/- SD, n=3 replicates (A) representative of two independent experiments (A,B).

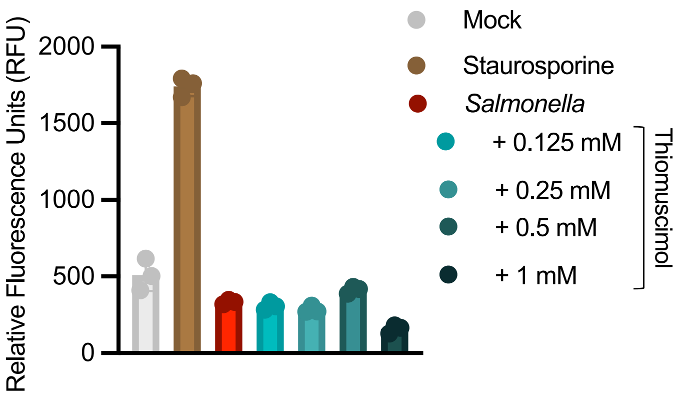

Supplemental Figure 2. Thiomuscimol does not redirect from pyroptosis to apoptosis. BMDM were infected with *Salmonella* in the presence of the indicated concentrations of thiomuscimol or induced to undergo apoptosis with staurosporine, and caspase-3 activity was assessed. Data are means +/- SD, n=3 replicates, representative of two independent experiments.

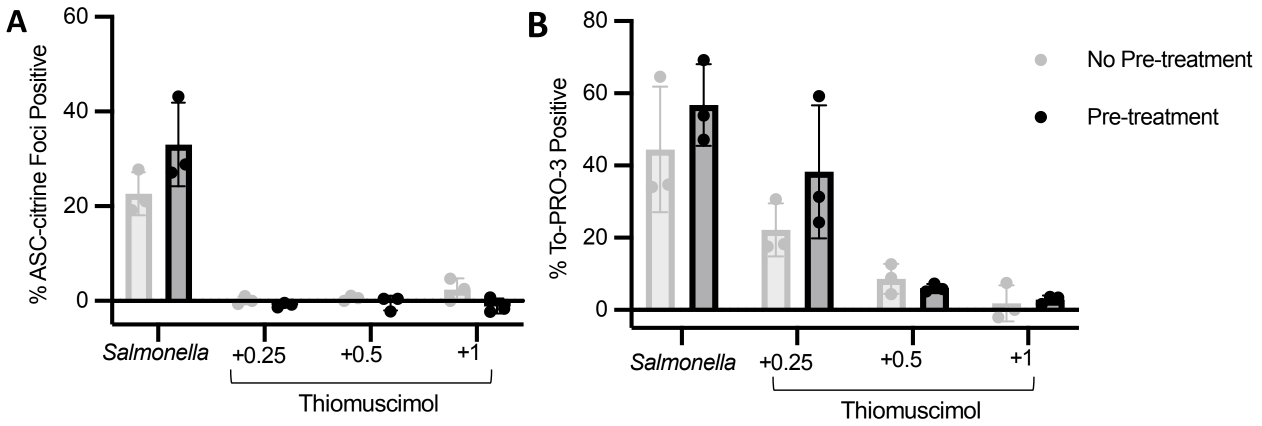

Supplemental Figure 3. Pretreatment does not significantly enhance thiomuscimol protection. ASC-citrine expressing BMDM were pretreated with thiomuscimol as indicated for one hour prior to infection with *Salmonella.* Localization of the inflammasome adapter ASC (yellow) and uptake of the small membrane-impermeant nuclear dye To-PRO-3 (red) were assessed one hour after infection (A-B). Data are means +/- SD, n=3 replicates, representative of two independent experiments.

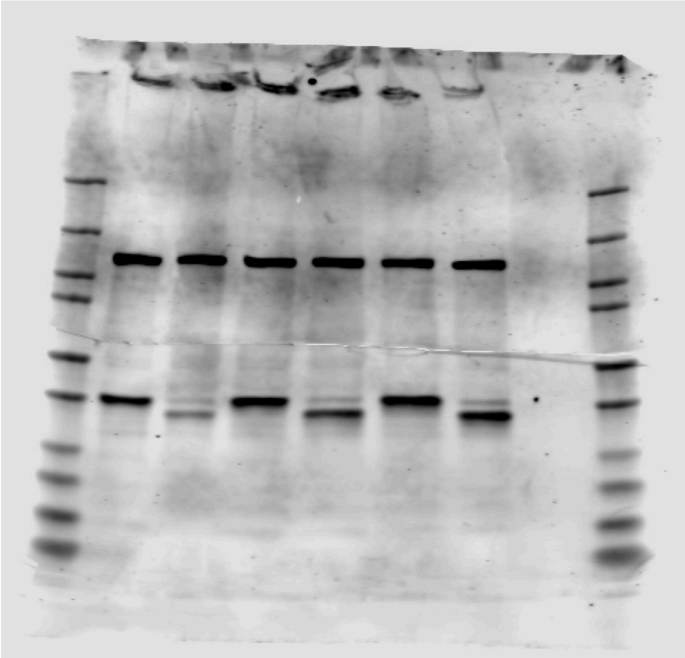

Supplemental Figure 4. Complete western blot
Uncropped western blot for supplemental figure 1.
